# Rate of intraoperative problems during sacroiliac screw removal: expect the unexpected

**DOI:** 10.1186/s12893-019-0501-0

**Published:** 2019-04-15

**Authors:** Georg Osterhoff, Jonas Noser, Kai Sprengel, Hans-Peter Simmen, Clément M. L. Werner

**Affiliations:** 1 Department of Trauma, University Hospital Zurich, Rämistrasse 100, 8091 Zurich, Switzerland; 2 Department of Orthopaedics, Trauma and Plastic Surgery, University Hospital of Leipzig, Liebigstr. 20, 04103 Leipzig, Germany

**Keywords:** Sacroiliac screw fixation, Iliosacral screw fixation, Transsacral screw fixation, Implant removal, Pelvic fracture, Sacral fracture, Sacroiliac joint

## Abstract

**Background:**

The indications for sacroiliac screw (SI) removal have been under debate. Data on complication rates of SI screw removal is missing in the current literature. The objective of this study was to compare the rate of intra- and perioperative problems and complications during SI screw removal to those with SI screw fixation.

**Methods:**

A retrospective observational study with two interventions in the same cohort was performed. Consecutive patients who underwent both sacroiliac screw fixation for an isolated fracture of the pelvic ring and removal of the same implants between November 2008 and September 2015 (*n* = 19; age 57.3, SD 16.1 years) were included. Intraoperative technical problems, postoperative complications, duration of surgery, and radiation dose were analysed.

**Results:**

Intraoperative technical problems occurred in 1/19 patients (5%) during SI screw fixation and in 7/19 cases (37%) during SI screw removal (*p* = .021). Postoperative complications were seen in 3/19 patients after SI screw fixation and in 1/19 patients after SI screw removal (*p* = 0.128). The surgical time needed per screw was longer for screw removal than for implantation (*p* = .005). The amount of radiation used for the whole intervention (*p* = .845) and per screw (*p* = .845) did not differ among the two interventions.

**Conclusions:**

Intraoperative technical problems were more frequent with SI screw removal than with SI screw fixation. Most of the intraoperative technical problems in this study were implant-related. They resulted in more surgical time needed per screw removed but similar radiation time.

## Background

Percutaneous sacroiliac (SI) screw fixation has become an established technique for posterior stabilization of pelvic ring injuries [1–6]. Complications associated with this surgical procedure include screw malposition, implant loosening, non-union, and damage to the L5 or sacral nerve roots [2, 7–13]. Revision rates between 2 and 19% were reported and in most cases the revision procedure was implant removal [2, 7–14].

In their case series on implant removal from the anterior and posterior pelvis, Stuby et al. [15] observed procedure-related complications in 20%.

On the topic of SI screw removal itself, there has been published only a small number of case reports and technical notes [16–19]. These stress the challenges associated with the intervention and point on the potential complications.

While implant removal may be well necessary in case of a symptomatic malpositioned screw, other indications for SI screw removal have been under debate. Some authors see an indication for removal only in case of complications [9, 20], others recommend implant removal routinely [7, 21]. Only about a third of the symptomatic patients report sufficient relief of their complaints after the procedure [15]. In addition, many patients ask for removal without being symptomatic.

To the authors’ knowledge, data on the complication rates of SI screw removal are underreported in the current literature. These would be important order to weigh the potential benefits against the risks associated with this procedure.

The purpose of this study was to compare the rate of intra- and perioperative problems and complications during SI screw removal to those to be expected with their implantation – in one and the same cohort of patients.

## Methods

The protocol of the present study was approved by the local ethics committee (Kantonale Ethik-Kommission Zürich, KEK-ZH-Nr. 2014–0557) and it was assured that patients included into this analysis had not objected to use of their data for research purposes.

A retrospective observational study was conducted. In order to avoid confounding factors as age, gender, co-morbidities, and the individual anatomy of the pelvis, a repeated measures design with two interventions in the same patient was chosen to compare the effect of two interventions (sacroiliac screw fixation and sacroiliac screw removal) on the perioperative outcome in the same patient. Consecutive patients who underwent both sacroiliac screw fixation for an isolated fracture of the pelvic ring and removal of the very same implants between November 2008 and September 2015 were identified (*n* = 19) from a pelvic ring fracture database (total number of patients with SI screw fixation in this period: *n* = 229) and a retrospective chart review was performed. Patients younger than 18 years and those with previous surgery to the pelvis were excluded.

### Pelvic screw fixation

Treatment decisions were based on the fracture classification described by Young & Burgess [22] using pelvic CT-scans and three-dimensional reconstructions thereof [23]. The decision for operative treatment was made in case of vertical shear (VS, OTA 61-C1) fractures, lateral compression (LC) type II (OTA 61-B2.2) and LC III (OTA 61-B3.2 and C2) fractures and of antero-posterior compression (APC) type III fractures (OTA 61-C1 and C3). For APC II (OTA 61-B1 and B3.1) fractures the indication for surgery was restricted to obese patients or patients that had to be mobilized quickly (i.e. elderly patients). In LC III and APC II and III injuries, anterior fixation was added by the means of plate fixation or internal subcutaneous fixation [24, 25]. Patients with LC I (OTA 61-B2.1) fractures were operated when non-operative treatment had failed or when a complete sacral fracture in addition with bilateral anterior ring injuries was present [26].

Surgery was performed with the patient supine using cannulated screws (7.3 mm; DePuy Synthes, Zuchwil, Switzerland) with a washer in the pedicles of S1 and/or S2 using conventional C-arm fluoroscopy as previously described [2, 4, 13]. In fractures without comminution, threads of 32 mm were placed in a lag screw fashion; otherwise, fully threaded screws were used.

Postoperatively, patients were allowed to mobilize with 15 kg partial weight bearing on the affected side for at least 6 weeks.

### Implant removal

Implant removal was performed in a short-term in-hospital setting. Patients with additional anterior implants underwent removal of the SI screws only. In all cases, it was aimed for a complete removal of the screws including washers. Through the scar of the previous surgery, a K-wire was introduced into the sacroiliac screw under C-arm assistance. The screw was then removed sliding a cannulated screw driver over the guide wire. The wire was left in place and served as a guide for either a hooked spoon or Kocher forceps in order to remove the washer. If this technique failed, a Kocher clamp, a nerve hook or an angulated scoop were directed towards the washer using the C-arm in different planes.

### Outcome parameter

Primary outcomes were intraoperative technical problems. These were defined as unexpected events that made additional measures necessary (e.g. opening an additional set of instruments) and prolonged the intervention by 10 or more minutes per screw. Secondary outcomes were postoperative complications during the time of hospitalization, these were categorized according to the system established by Clavien and Dindo [27]. Malpositioned screws that were detected during the initial intervention were revised immediately. Malpositioned screws that were detected postoperatively were defined as a postoperative complication. A symptomatic malpositioned screw was defined as a screw that is not placed within the planned bony canal but breaches the cortical borders of the sacrum and causes pain or loss of motor or sensory function.

Tertiary outcomes were duration of surgery and radiation dose.

### Statistical analysis

Statistical analysis was done by the use of SPSS for windows 22.0 (SPSS, Chicago, Illinois, USA). Ordinal and metric data was compared using non-parametric tests; associations between categorical data were tested using crosstabs and Fisher’s Exact test. Metric data was presented as mean with standard deviation (SD). Differences were considered significant for values of *p* < 0.05. In case of missing data, comparisons were made only with the data available and this was reported in the results section accordingly.

## Results

### SI screw fixation

Nineteen patients (age 57.3, SD 16.1 years, range 22 to 86 years; 13 female) were included into the final analysis. The indication for sacroiliac screw fixation were traumatic fractures in 17 cases (Young & Burgess: 14 LC I, 2 LC II, 1 APC II, 1 combined mechanism) and sacral fragility fractures (2 H-type) in two cases. The total number of screws was 4 screws in 2 patients, 3 screws in 1 patient, 2 screws in 8 patients and 1 screw in 8 patients. Hence, the average number of screws inserted was 1.8 (SD 1.0). Twelve received unilateral and 7 patients a bilateral fixation. In all patients, at least one screw was placed into S1, while 9 patients received an additional screw into S2. In one patient with two bilateral screws in S1 and two bilateral screws in S2, the screws in S1 were augmented with PMMA cement.

### SI screw removal

Sacroiliac screw removal in the same patients was performed mean 213 days (SD 194, range 2 to 606 days) after implantation of the screws. The indication for screw removal was screw malposition in three cases (see also postoperative complications during hospitalization listed below), asymptomatic screw loosening in five cases, SI joint pain in five cases and a planned pregnancy in one case. In five additional asymptomatic cases, the screws were removed only on the patients’ explicit request.

### Outcome

Intraoperative technical problems occurred in 1/19 patients (5%) during SI screw fixation. The patient had an unexpected accumulation of bowel gas that prevented safe placement of an S2 screw.

**Fig. 1:**
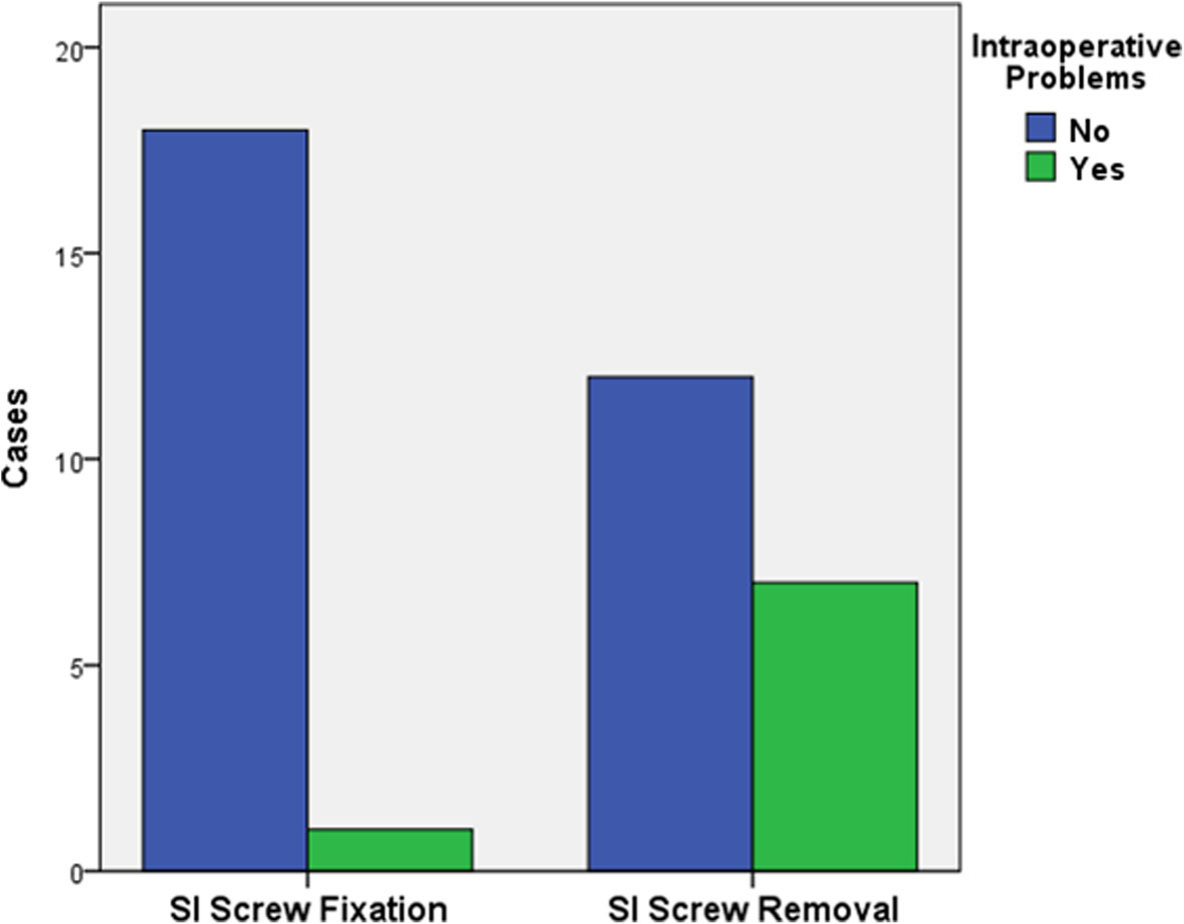
Intraoperative technical complications

Complete SI screw removal including the washers was achieved in all patients. No broken screws were observed. During SI screw removal, additional intra-operative measures were necessary in 7/19 cases (37%; *p* = .021, Fig. 1). These included problems due to osseous ingrowth of the washer (*n* = 4), the need for an extended incision due to ingrowth of the screw (*n* = 2), and one case with a worn out screw head. A post-hoc power analysis for the primary outcome (intraoperative technical problems) revealed a power of 1.0 based on an observed odds ratio of 10.5 and assuming a correlation coefficient (ϕ) for exposure between matched cases and controls of 0.2 [28].

**Fig. 2:**
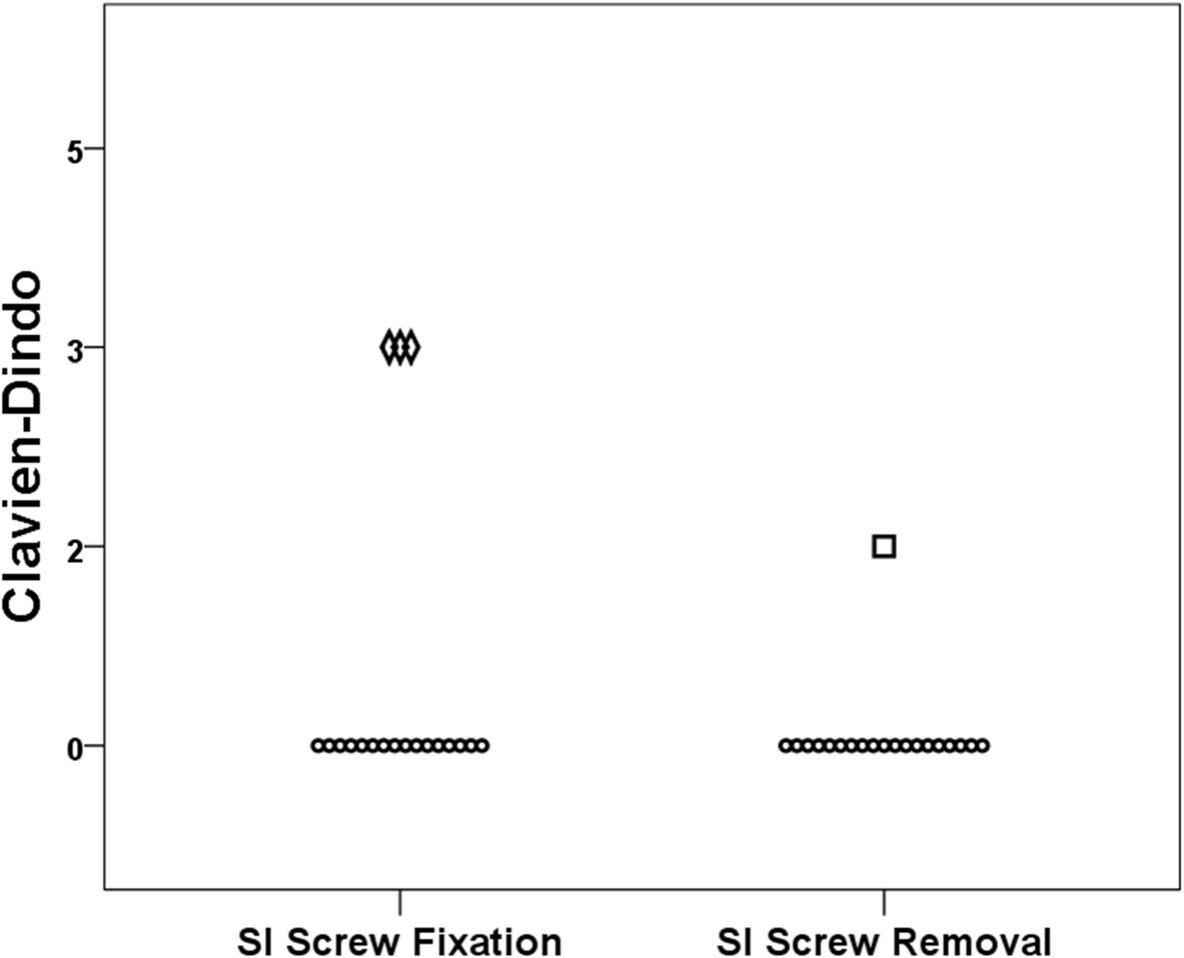
Postoperative complications. Each symbol represents one patient

Postoperative complications during the time of hospitalization were seen in three patients after the initial SI screw fixation procedure and in one patient after SI screw removal. After screw fixation, three patients had to undergo revision for screw malposition. After screw removal, one patient had a urinary tract infection that was successfully treated with antibiotics. When categorized according to the classification described by Clavien and Dindo, there were no significant differences among the two groups (*p* = 0.128, Fig. 2).

While the total duration of surgery was not significantly different (*p* = .075) among the two groups, the time needed per screw was longer for screw removal than for implantation (*p* = .005, Table 1).

**Table 1:** Duration of surgery and radiation dose

	N	SI Screw Fixation	Removal	*p*
Duration of surgery [min]	19			
total		29.0 (11.4)	37.6 (14.8)	.075
per screw		15.2 (5.3)	21.3 (6.3)	.005
Radiation dose [mGy]	13			
total		27.2 (19.3)	27.5 (21.2)	.845
per screw		11.4 (6.5)	13.0 (8.6)	.845

Data reported as mean and standard deviation (in brackets)

*min* minutes, *mGy* Milli-Gray

Complete data regarding radiation dose was available in only 13 patients. For these, the amount of radiation used for the whole intervention (*p* = .845) and per screw (*p* = .845) did not differ among the two interventions (Table 1).

## Discussion

There has been existing uncertainty on the need and benefit of SI screw removal in cases other than for screw malpositioning or implant failure [7, 9, 15, 20, 21]. The purpose of this study was to evaluate intra- and perioperative problems during SI screw removal and to compare these with the initial procedure of SI screw fixation. In our series of 19 patients, we observed more intraoperative technical problems with SI screw removal than with SI screw fixation. These resulted in more surgical time per screw needed for removal than for implantation.

The rate of intraoperative technical problems was consistent with data from a series on implant removal from the anterior and posterior pelvis, that reported intraoperative problems with the washer or broken screws in 4/19 cases with SI screw removal [15].

The postoperative complication rates for SI screw fixation (19%) seen in our study population were higher than those reported in the literature [7–11]. In contrast to other series, however, our study included only patients who had undergone both SI screw fixation and removal. This may have skewed patient selection towards those with malpositioned screws requiring screw removal. Misplaced screws were classified as postoperative complications if they required surgery only. One could argue that these are intraoperative complications of the index surgery. However, if the surgeon does not identify them during surgery they only become a complication if the patient suffers pain or neurological deficits. Otherwise, they are simply radiographically misplaced screws without clinical relevance.

The number of postoperative complications after SI screw removal was smaller than what would be expected from the small body of literature that reports complication rates of about 20% with implant removal after pelvic fracture stabilization [15].

It could be shown, that SI screw removal is technically challenging and may require more OR time than screw implantation.

Most of the intraoperative technical problems in this study were implant-related. A potential solution to decrease implant-related intraoperative problems is the use of advanced operative techniques. Described surgical techniques to address the difficulties associated with the retrieval of the washer include endoscopic techniques [18] or the use of a tap of larger diameter in order to achieve interference fit [19]. Broken screws can be removed by using a push screw or drill from the contra-lateral side if this is possible by the screw’s orientation [16].

Removing the washer seemed to be the part of the procedure that was most prone to problems. There would have been less intraoperative technical problems and less radiation exposure if we had simply left the washer inside the patients. In fact, without the problems caused by washers, the differences between the two interventions would have been negligible. Just removing the screw and leaving the washer where it is, is a frequent topic that we discuss with the patients preoperatively. However, most of the patients preferred complete implant removal and thus leaving the washer may be not a good option in these cases. In contrast, a prolonged surgical time or even an extended incision with additional surgical risk to remove a washer that is asymptomatic at the preference of the patient might be an even less preferable option.

A different approach to decrease implant-related problems is the development of better implants. While the biomechanical effect of using washers to allow for better compression has been shown [29], their removal obviously is associated with difficulties. One solution could be a washer that is movably mounted to the screw head and, hence, would allow for removal of the screw and the washer as a whole.

The limitations of this study are related to its retrospective study design and the small number of patients included. Even though the matched or repeated measure design of this study allows for ruling out age, gender, co-morbidities, and the individual anatomy of the pelvis as confounding factors, it is not possible to finally conclude that SI screw removal is more prone to complications than SI screw fixation. The time of screw removal ranged from 2 days to almost 2 years. This may have caused a bias with regard to more osseous overgrowth of the screw and washer in patients with late removal. With the numbers available, however, further subgroup analyses were not possible and could be objective of future studies.

The data for radiation exposure was incomplete (13/19). However, radiation exposure was not the primary outcome but chosen as an additional secondary parameter in order to quantify the number of fluoroscopic images taken intraoperatively and to illustrate the surgeon’s strain.

Further studies are needed to prove any potential benefit of SI screw removal in patients other than those with complications as symptomatic implant malpositioning. Patients who wish their implants to be removed should meticulously be informed on the risks and benefits of this procedure.

## Conclusions

Intraoperative technical problems were more frequent with SI screw removal than with SI screw fixation. Most of the intraoperative technical problems in this study were implant-related. They resulted in more surgical time needed per screw removed but similar radiation time.

In light of our results, SI screw removal is an intervention with considerable radiation exposure and high rates of intraoperative problems. Even though the complications associated with this procedure rarely have long-term consequences, routine removal cannot be recommended.

## Abbreviations

APC: Anterior-posterior compression
CT: Computed tomography
LC: Lateral compression
SD: Standard deviation
SI: Sacroiliac
